# Supplementary figures and images for: Shared behavioural impairments in visual perception and place avoidance across different autism models are driven by periaqueductal grey hypoexcitability in Setd5 haploinsufficient mice
Source: PLoS Biol. 2024 Jun 10;22(6):e3002668. doi: 10.1371/journal.pbio.3002668 (PMC11216578; doi:10.1371/journal.pbio.3002668)

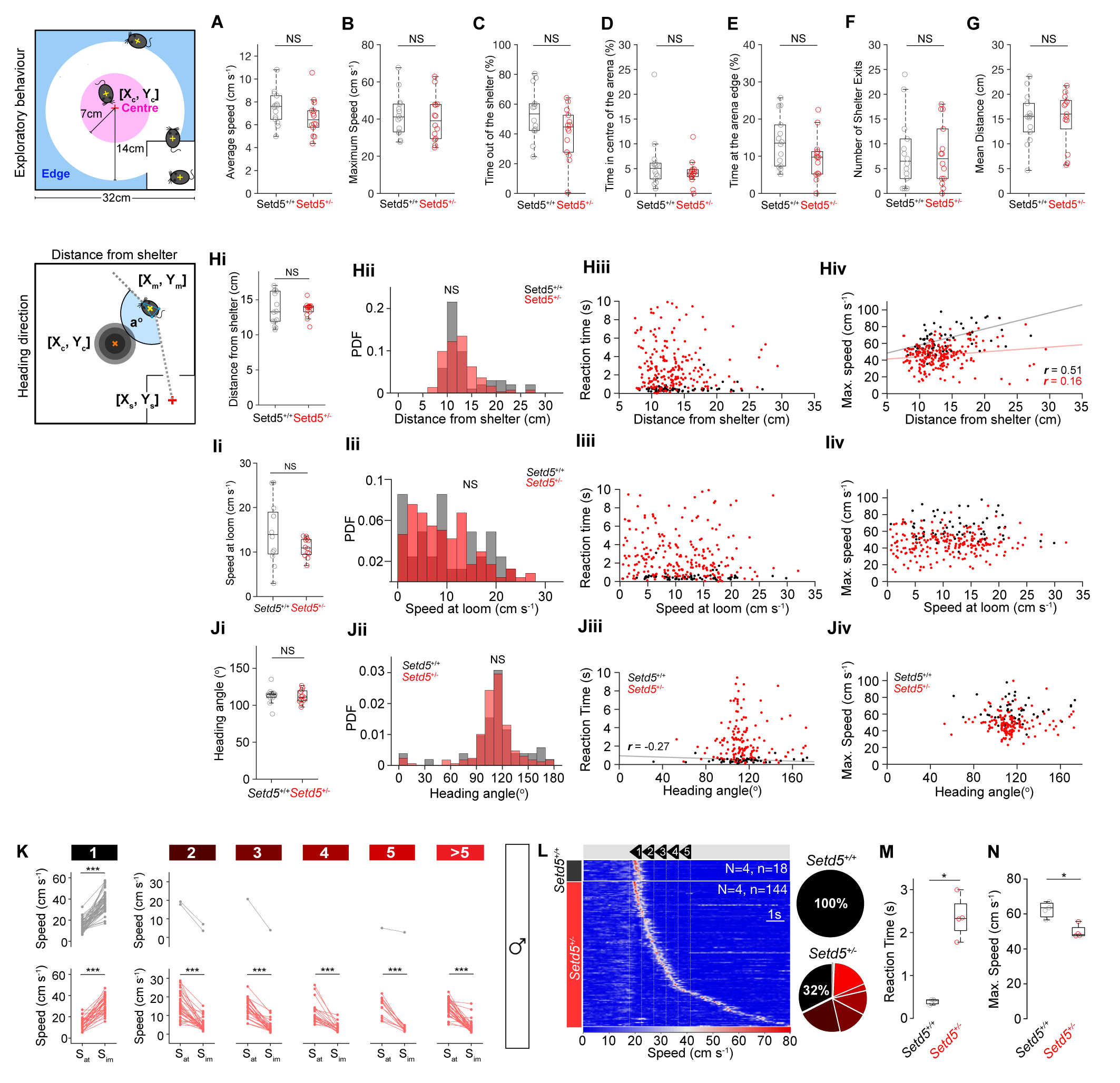

Supplement: S1 Fig — No significant difference in the exploratory behaviour of the Setd5+/+ and Setd5+/− animals during the prestimulus exploration. The top graphic depicts the dimensions and regions of the arena that are classified as the centre (pink, a circular region with a radius of 7 cm from the centre of the arena [Xc,Yc]), and the edge (blue, the area outside of a circular area with a 14-cm radius from [Xc, Yc]). (A) Average speed during prestimulus exploration (Setd5+/+, 7.50 cm s−1; Setd5+/−, 6.65 cm s−1, P = 0.151). (B) Maximum speed (Setd5+/+, 42.2 cm s−1; Setd5+/−, 40.2 cm s−1, P = 0.642). (C) Time spent out of the shelter (Setd5+/+, 53.5%; Setd5+/−, 40.9%, P = 0.062). (D) Time spent in the centre of the arena (Setd5+/+, 6.00%; Setd5+/−, 4.20%, P = 0.448). (E) Time spent at the edge of the arena (Setd5+/+, 13.9%; Setd5+/−, 9.47%, P = 0.076). (F) Number of shelter exits (Setd5+/+, 8.14; Setd5+/−, 7.88, P > 0.999). (G) Mean distance travelled during exit (Setd5+/+, 15.2 cm; Setd5+/−, 15.1 cm, P = 0.981). The lower graphic depicts the distance of the mouse from the shelter as the Euclidean distance between the centre of mouse, [Xm, Ym], and the centre of shelter, [Xs, Ys], and the heading angle as the angle (a°) between a line drawn along the body axis of the mouse and another from the position between the back paws of the mouse and the centre of the shelter. The bottom graphic depicts how the directedness of the escape trajectory is calculated by finding the shortest Euclidean distance between the centre of the mouse and the edge of the shelter when the stimulus starts and the actual distance travelled between the stimulus start and the mouse reentering the shelter. (Hi) Median distance from the shelter when the looming stimulus is triggered (Setd5+/+, 13.2 cm; Setd5+/−, 13.7 cm, P = 0.341). (Hii) Distribution of distances from the shelter across all trials, pooled by genotype (p = 0.325, two-way Kolmogorov–Smirnov test). (Hiii) Relationship between the distance to the shelte [file pbio.3002668.s001.tif]

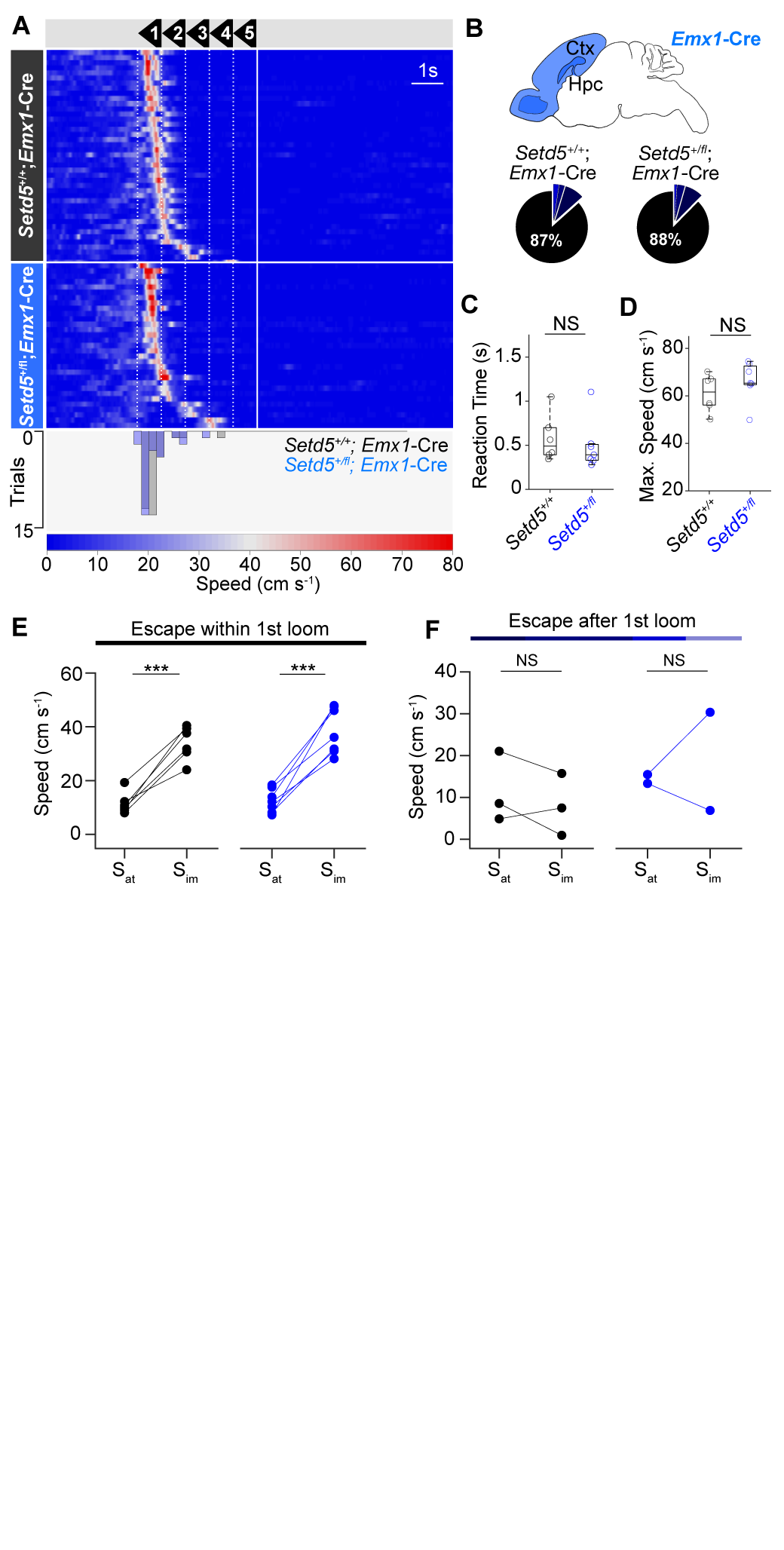

Supplement: S2 Fig — (A) Raster plot of mouse speed in response to the looming stimuli (white, dotted vertical lines denote the start of each loom; white solid line denotes the end of the stimulus) for Setd5+/+; Emx1-Cre (top, n = 6, 42 trials) and Setd5+/fl; Emx1-Cre (bottom, n = 6, 32 trials), sorted by reaction time. Bottom, distribution of number of looms to escape across all trials (Setd5+/+; Emx1-Cre, 32 trials, Setd5+/fl;Emx1-Cre, 42 trials, p = 0.960, two-sample Kolmogorov–Smirnov (KS) test). (B) Pictorial representation of the expression of Emx1 in a sagittal section of a mouse brain, showing the localisation of the expression to the cortex and hippocampus (blue, top). Proportion of trials in which the mice respond within each loom for Setd5+/+; Emx1-Cre (middle) and Setd5+/fl; Emx1-Cre mice (bottom). (C) Reaction times and (D) maximum escape speed (Setd5+/+; Emx1-Cre, n = 6, 61.1 cm s−1; Setd5+/fl;Emx1-Cre, n = 6, 66.4 cm s−1, P = 0.295). (E) Speed immediately following the stimulus presentation for trials where the mice escape within the first loom presentation (left, Setd5+/+; Emx1-Cre, n = 6, p < 0.001; right, Setd5+/fl;Emx1-Cre, n = 6, p < 0.001). (F) Speed immediately following the stimulus presentation for trials where the mice escape after the first loom presentation (left, Setd5+/+, n = 3, p = 0.6248, paired t test; right, Setd5+/−, n = 2, p = 0.7546). P-values: Wilcoxon’s ranked-sum test. p-values: two-tailed paired t test, unless specified. The data underlying this figure can be found in S10 Data. (TIF) [file pbio.3002668.s002.tif]

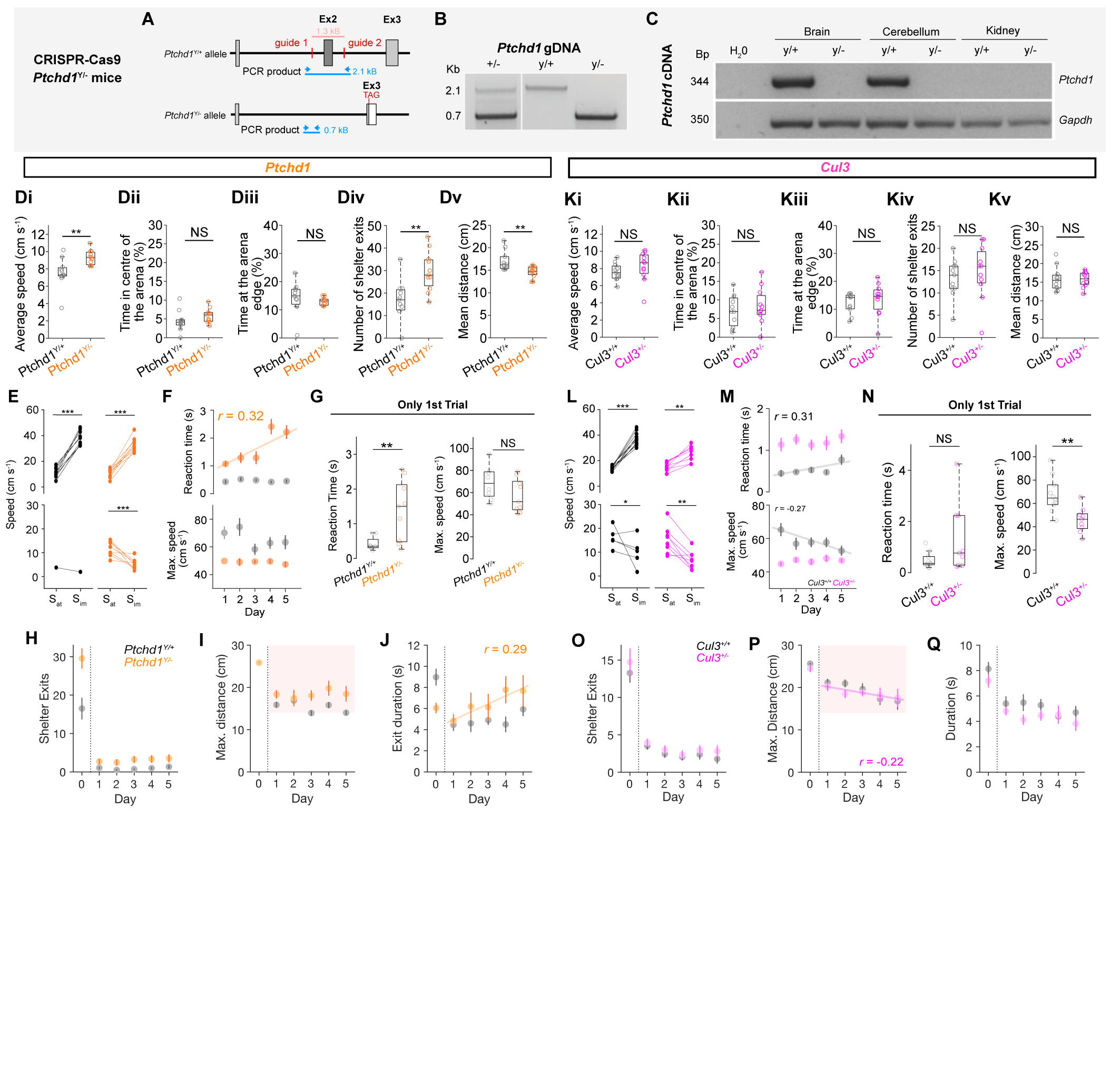

Supplement: S3 Fig — (A) CRISPR-Cas9 design for the generation of Ptchd1Y/− mice. (B) gDNA confirmation of the excised DNA (2.1 Kb in Ptchd1Y/+ and 0.7 Kb in Ptchd1Y/−). (C) Confirmation of loss of Ptchd1 cDNA in different tissues in Ptchd1Y/− mice, and control Gapdh presence throughout. (D) Exploration controls for Ptchd1. (Di) Average speed during prestimulus exposure acclimatisation (Ptchd1Y/+, 7.53 cm s−1; Ptchd1Y/−, 9.31 cm s−1, p = 0.003). (Dii) Time spent in the centre of the arena (Ptchd1Y/+, 4.46%; Ptchd1Y/−, 5.86%, p = 0.170). (Diii) Time spent at the edge of the arena (Ptchd1Y/+, 14.3%; Ptchd1Y/−, 13.0%, p = 0.148). (Div) Number of shelter exits (Ptchd1Y/+, 17.0, Ptchd1Y/−, 29.5, p = 0.003). (Dv) Average distance travelled during exit (Ptchd1Y/+, 17.1 cm, Ptchd1Y/−, 14.6 cm, p = 0.004). (E) Speed change immediately following the stimulus presentation for trials where the mice escape within the first loom presentation (top left, Ptchd1Y/+, n = 9, black, p < 0.001, two-tailed t test; top right, Ptchd1Y/−, n = 9, red, p < 0.001, two-tailed t test). Speed change immediately following the stimulus presentation for trials where the mice escape after the first loom presentation (bottom left, Ptchd1Y/+, n = 1, black; bottom right, Ptchd1Y/−, n = 8, red, p < 0.001, two-tailed t test). Sat is the mean speed of the animal ±50 ms of stimulus onset, and Sim is the mean speed of the animal 300-800ms after stimulus onset. (F) Top, relationship between reaction time and test day (Ptchd1Y/+, r = 0.029, p = 0.840; Ptchd1Y/−, r = 0.317, p < 0.001) and bottom, maximum escape speed and test day (Ptchd1Y/+, r = −0.254, p = 0.07; Ptchd1Y/−, r = −0.040, p = 0.556). (G) Mean reaction time (left) and maximum escape speed (right) per animal, for the very first loom presentation (reaction time; Ptchd1Y/+, 0.423 s, Ptchd1Y/−, 1.39 s, P = 0.014; maximum escape speed; Ptchd1Y/+, 69.1 cm s−1, Ptchd1Y/−, 57.3 cm s−1, P = 0.139). (H-J) Relationship between test day and the number of exits from shelter (H, Ptc [file pbio.3002668.s003.tif]

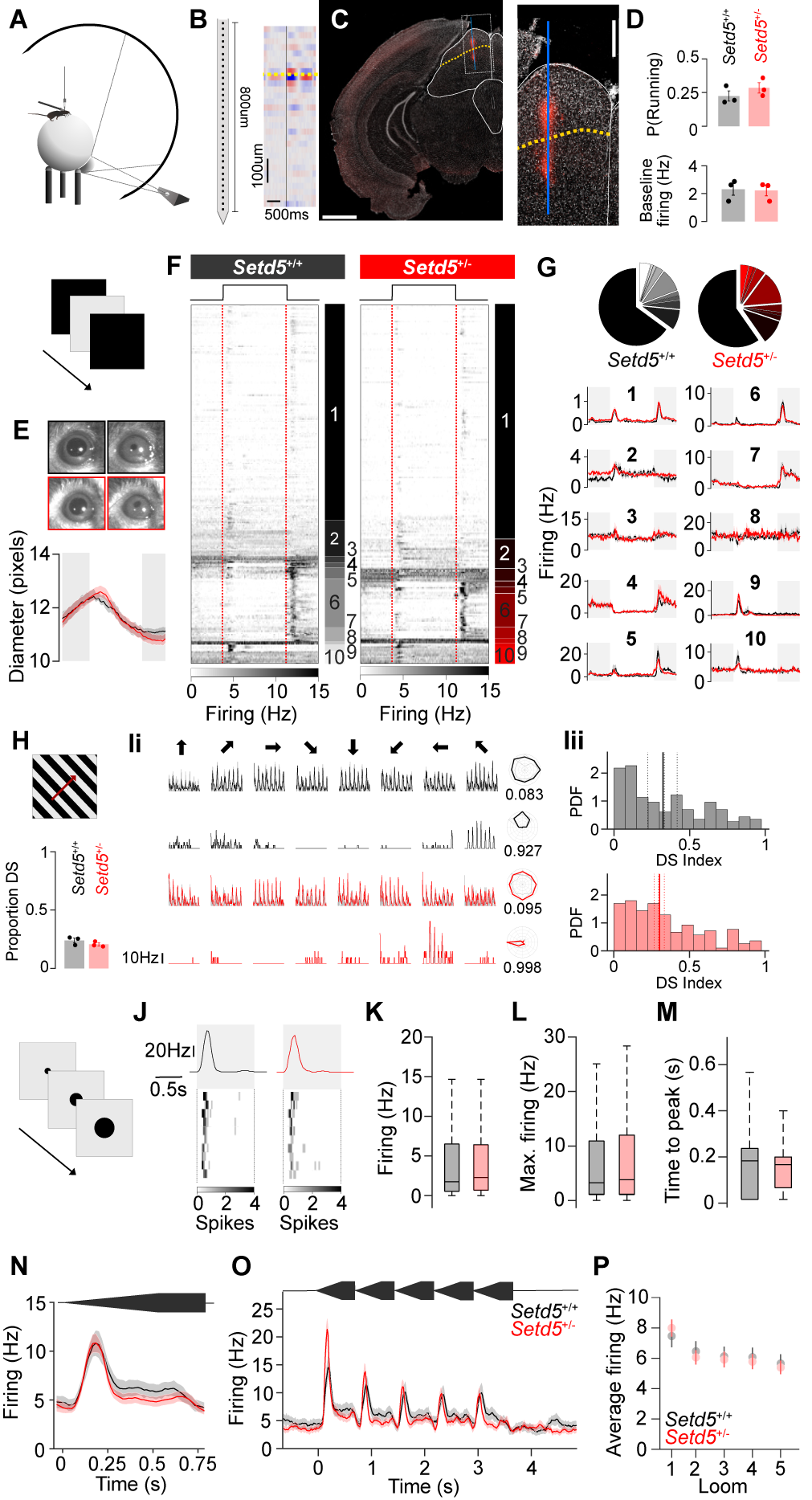

Supplement: S4 Fig — (A) Schematic of the in vivo recording setup displaying the spherical treadmill and light projector illuminating the spherical screen. (B) Left, schematic of the 32-channel silicon probe used to record extracellular activity aligned with the current source density analysis of a single flash stimulus averaged over 10 presentations. Black vertical line represents the stimulus onset; yellow dotted line marks the inflection depth separating the current source and sink. (C) Histological reconstruction of the probe position marked with DiI (120 μm coronal section, scale bar: 1 mm) with a close-up (scale bar: 250 μm). Yellow dotted line as in (B). (D) Proportion of time the mice were moving during the recording sessions (P = 0.400, top) and SC’s baseline firing rate (P = 0.880, bottom). (E) Pupil dilation in response to the full-field flash stimulus. Example images of dilated and constricted pupils in responses to the OFF (left) and ON (right) periods of the flash. Setd5+/+ (black), Setd5+/− (red) (P = 0.060). (F) Sorted raster plot of neural responses to a single flash stimulus. Vertical red dotted lines indicate the on and offset of the flash stimulus. (G) Proportion of cells in each cluster (top). Mean ± SEM of the flash responses in each cluster (traces). (H) Proportion of direction-selective SC cells (see Materials and methods) to full field gratings (Setd5+/+, 87 of 153 units, 0.287; Setd5+/−, 159 of 259 units, 0.2432, P = 0.322, Wilcoxon’s test). (I_i) Firing rate of example units in response to full field gratings moving in 8 different directions for Setd5+/+ (black) and Setd5+/− (red) with a summary polar plot of their direction selectivity (right) and the corresponding direction selectivity index (DSI) value. Bold lines show the mean response across 3 repetitions (light grey lines). (I_ii) Distribution of DSI across SC units (top, Setd5+/+, 153 units, median DSI = 0.323; bottom, Setd5+/−, 259 units, median DSI = 0.3018, P = 0.7054, two-sample Kolmogorov–Smirnov t [file pbio.3002668.s004.tif]

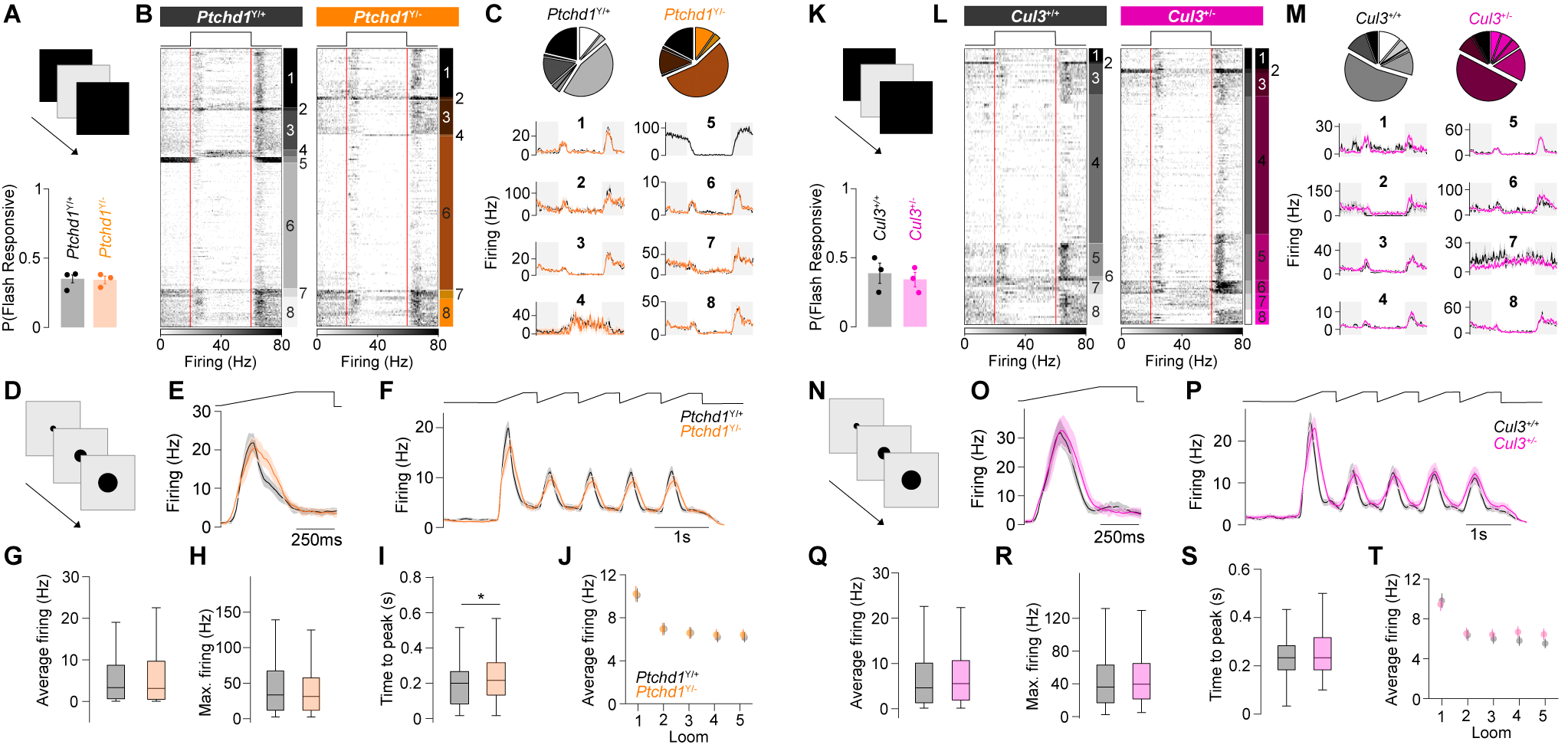

Supplement: S5 Fig — (A) Schematic of visual stimulus and proportion of sSC cells that are flash responsive (Ptchd1Y/+: 0.350; Ptchd1Y/−: 0.343, P = 0.857, two-way t test). (B) Sorted raster plot of neural responses to a single flash stimulus. Vertical red dotted lines indicate the onset and offset of the flash stimulus. (C) Proportion of cells in each cluster (top). Mean ± SEM of the flash responses in each cluster (traces). (D) Schematic of the loom stimulus. (E) Mean ± SEM response of all of the first loom stimuli and (F) of the 5 consecutive loom stimuli for Ptchd1Y/+ (black) and Ptchd1Y/− (orange) sSC units. (G-I) Summary of mean, maximum, and time-to-peak firing (G, P = 0.461; H, P = 0.523; I, P = 0.049). (J) Average firing to a single loom across the 5-loom stimulus (p = 0.758). (K-T) As for (A-J) but for the Cul3 mouse model. (K) Proportion of sSC cells that are flash responsive (Cul3+/+: 0.388; Cul3+/−: 0.342, P = 0.638, two-way t test). (L) Sorted raster plot of neural responses to a single flash stimulus. Vertical red dotted lines indicate the onset and offset of the flash stimulus. (M) Proportion of cells in each cluster (top). Mean ± SEM of the flash responses in each cluster (traces). (N) Schematic of the loom stimulus. (O) Mean ± SEM response of all of the first loom stimuli and (P) of the 5 consecutive loom stimuli for Cul3+/+ (black) and Cul3+/− (magenta) sSC units. (Q-S) Summary of mean, maximum, and time-to-peak firing (Q, P = 0.222; R, P = 0.214; S, P = 0.879). (T) Average firing to a single loom across the 5-loom stimulus (p = 0.267). Ptchd1Y/+, n = 3, 263 sSC cells; Ptchd1Y/−, n = 3, 202 sSC cells. Cul3+/+, n = 3, 205 sSC cells; Cul3+/−, n = 3, 204 sSC cells. P-values: Wilcoxon’s test, p-values: two-way repeated measures ANOVA. The data underlying this figure can be found in S14 Data. (TIF) [file pbio.3002668.s005.tif]

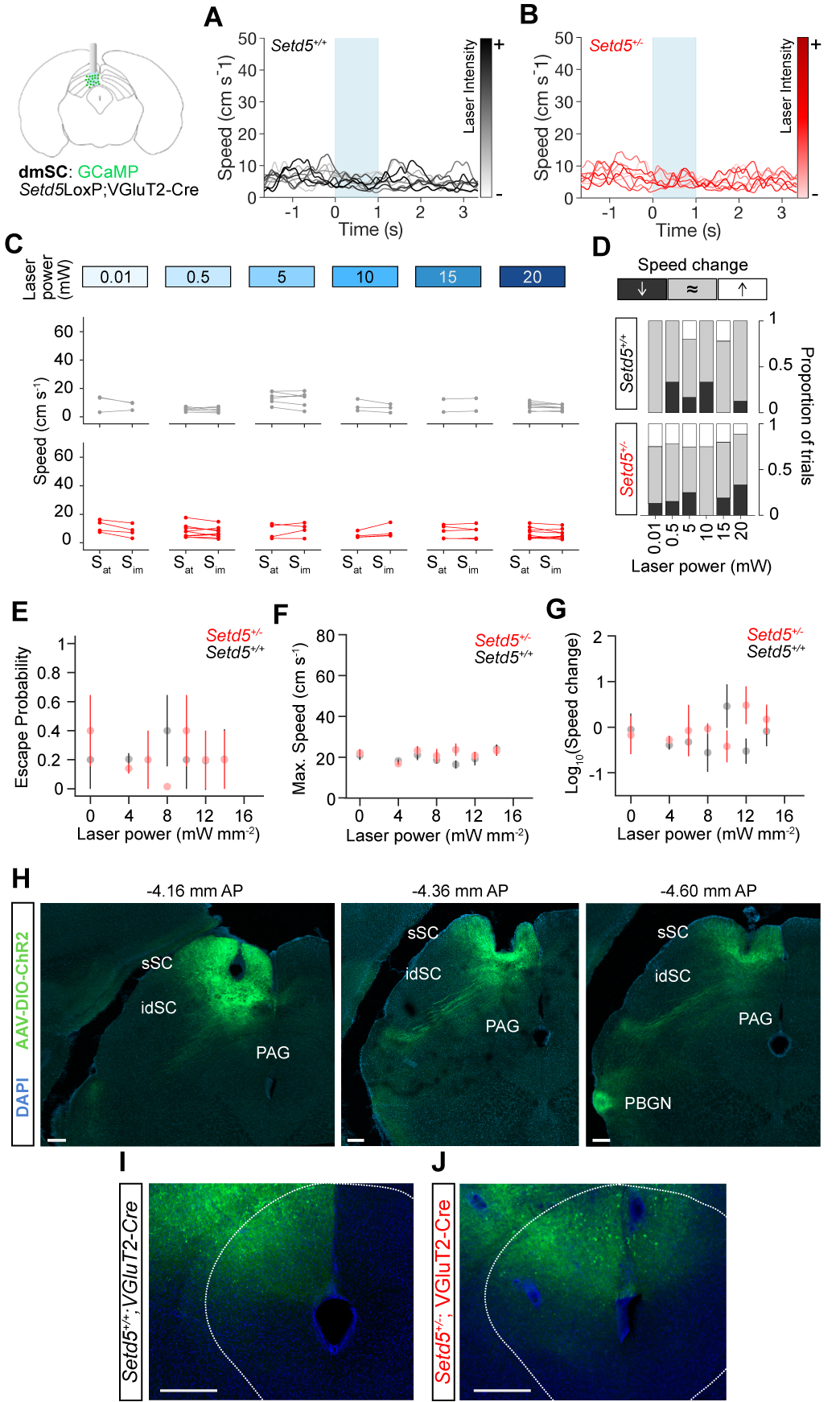

Supplement: S6 Fig — (A, B) Mean speed responses to light stimulation at increasing laser intensities in Setd5+/+;VGluT2-Cre (A) and Setd5+/−;VGluT2-Cre (B) mice injected with AAV-GCaMP6m. Blue shaded areas show 1 s of 10 Hz light stimulation. (C) Change in speed upon light activation at different laser intensities for Setd5+/+ (top, n = 1. 0.01 mW mm−2: P = 0.700, 3 trials; 0.5 mW mm−2: P = 0.472, 10 trials; 5.0 mW mm−2: P > 0.995, 5 trials; 10.0 mW mm−2: P = 0.700, 3 trials; 15.0 mW mm−2: P = 0.667, 2 trials; 20.0 mW mm−2: P = 0.442, 8 trials) and Setd5+/− (bottom, n = 1. 0.01 mW mm−2: P = 0.343, 4 trials; 0.5 mW mm−2: P = 0.796, 9 trials; 5.0 mW mm−2: P = 0.279, 4 trials; 10.0 mW mm−2: P = 0.200, 4 trials; 15.0 mW mm−2: P = 0.887, 4 trials; 20.0 mW mm−2: P = 0.678, 10 trials) trials. Sat is the mean speed of the animal ±50 ms of laser onset, and Sim is the mean speed of the animal 300-800ms after laser onset. (D) Proportion of trials where the speed of the mouse increases (white: Sim > Sat by more than 1 SD), decreases (black: Sim < Sat by more than 1 SD) or does not change (grey: Sim less than 1 SD different from Sat). 0.01 mW mm−2: 7 trials, p = 0.165; 0.5 mW mm−2: 19 trials, p = 0.624; 5 mW mm−2: 9 trials, p = 0.852; 10 mW mm−2: 7 trials, p = 0.766; 15 mW mm−2: 6 trials, p = 0.349; 20 mW mm−2: 18 trials, p = 0.815, X2 test of independence. (E) No relationship between escape probability and laser power (Setd5+/+, r = −0.005, p = 0.955; Setd5+/−, r = −0.047, p = 0.577). (F) No relationship between maximum speed and laser power (Setd5+/+, r = −0.014, p = 0.871; Setd5+/−, r = 0.011, p = 0.8986). (G) No relationship between the Log10(Immediate ΔSpeed) and laser power (Setd5+/+, r = −0.057, p = 0.496; Setd5+/−, r = 0.283, p = 0.149). (H) Confocal micrographs of AAV-DIO-ChR2 viral expression pattern at different anterior-posterior (AP) positions and optic fibre placement above the idSC, coordinates are in mm and from bregma. (I, J) Close-up to the dPAG in Setd5+/+ and Setd5+/−, respectiv [file pbio.3002668.s006.tif]

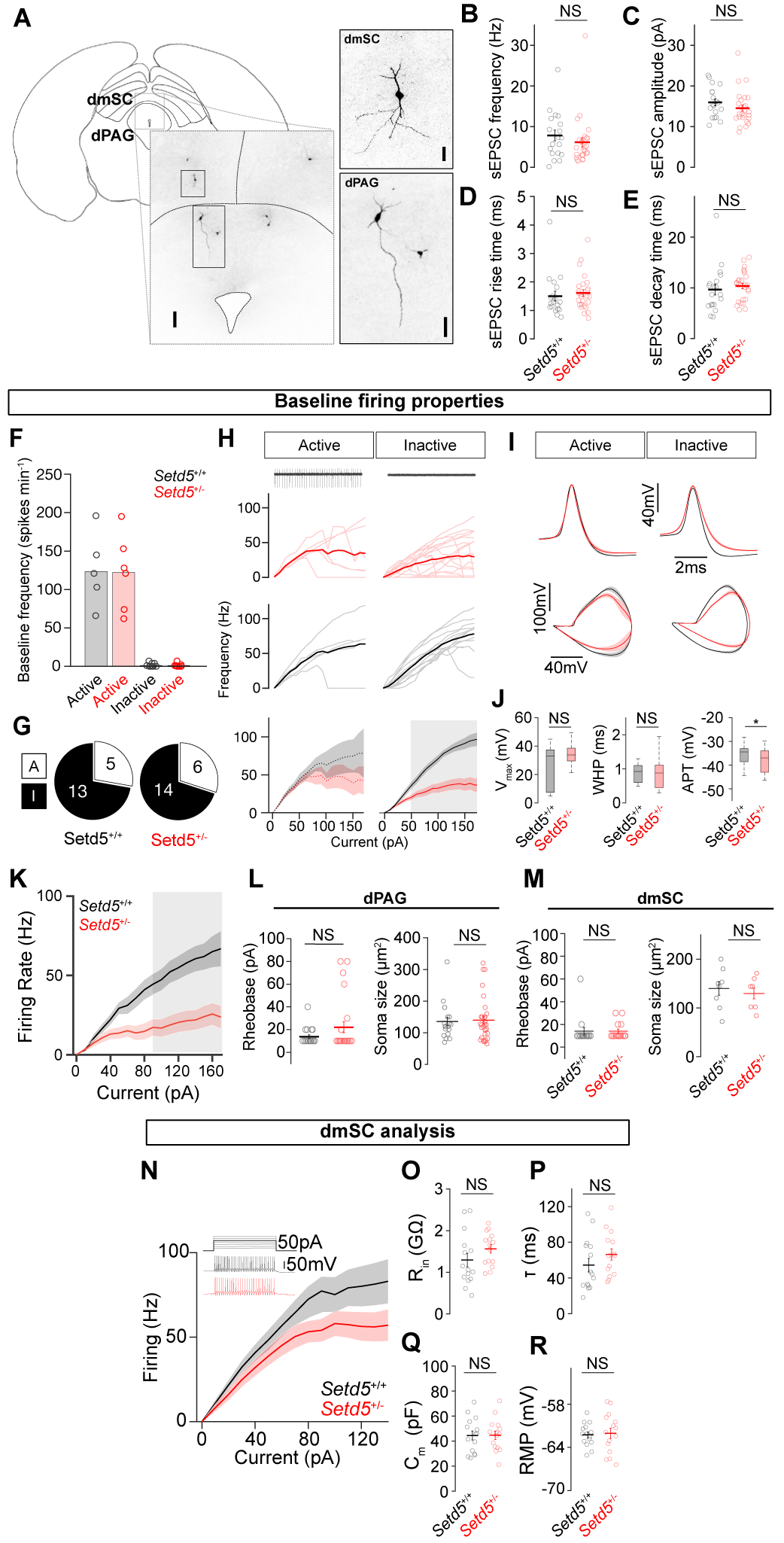

Supplement: S7 Fig — (A) Location of dPAG and dmSC cells that were recorded and filled with biocytin (scale bar: 100 μm). Insets show close-ups of the biocytin-filled dPAG cells (top, scale bar: 50 μm) and dmSC cells (bottom, scale bar: 20 μm). (B-E) Spontaneous neurotransmission in dPAG (Setd5+/+: n = 6, 19 cells; Setd5+/−: n = 7, 21 cells). (B) Average sEPSC frequency (Setd5+/+: 7.8 Hz; Setd5+/−: 5.0 Hz, P = 0.7743); (C) average sEPSC amplitude (Setd5+/+: 16.0 pA; Setd5+/−: 14.0 pA, P = 0.704); (D) average sEPSC rise time (Setd5+/+: 1.50 ms; Setd5+/−: 1.60 ms, P = 0.948); (E) average sEPSC decay time (Setd5+/+: 9.64 ms; Setd5+/−: 10.5 ms, P = 0.838). (F) Firing frequency at baseline splits the recorded cells into 2 groups. (Average baseline frequency: Setd5+/+ high frequency, 5 cells, 123.5 Hz; Setd5+/− high frequency, 6 cells, 122.2 Hz; Setd5+/+ low frequency, 13 cells, 1.12 Hz; Setd5+/− low frequency, 14 cells, 1.00 Hz). (G) Proportion of recorded cells from Setd5+/+ (left) and Setd5+/− (right) mice that exhibit high frequency (active) or inactive at baseline. (H) Top, example traces from an active (high frequency firing), putative GABAergic, cell (left) and an inactive, putative glutamatergic, cell (right) at baseline. Centre top, mean current-firing relationship of all putative GABAergic (left) and putative glutamatergic (right) Setd5+/− cells in red. Light pink lines represent individual cells. Centre bottom, mean current-firing relationship of all putative GABAergic (left) and putative glutamatergic (right) Setd5+/+ cells in black. Light grey lines represent individual cells. Bottom, summary of the relationship between current injection and action potential firing for putative GABAergic (left, P = 0.560 for the effect of genotype) and glutamatergic (right, P < 0.001 for the effect of genotype) cells. The grey area indicates the current injection values that are significantly different between Setd5+/+ cells (black) and Setd5+/− cells (red) found by a multiple comparisons analysi [file pbio.3002668.s007.tif]

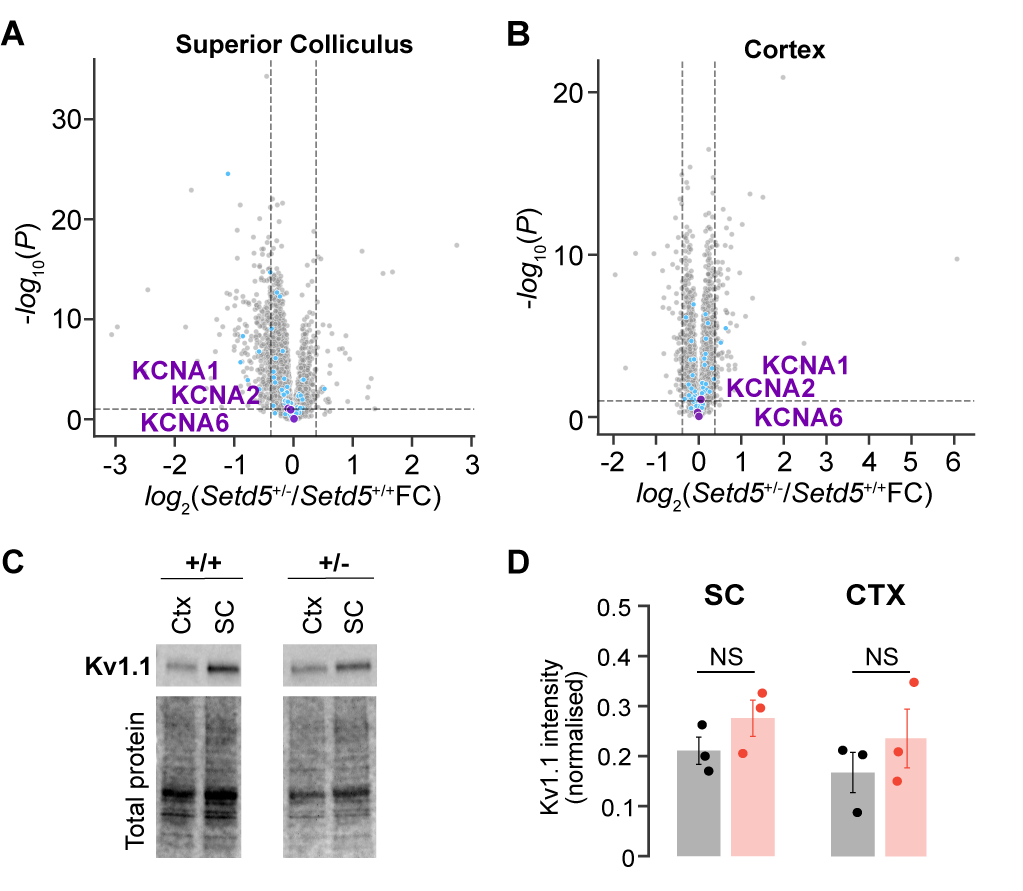

Supplement: S8 Fig — (A, B) Volcano plots for differential protein levels in the superior colliculus (SC) and cortex (CTX) between adult Setd5+/+ and Setd5+/− mice (n = 6 independent samples per genotype and brain area, cyan dots represent annotated proteins as ion-channels, and purple dots represent Kv1.1, Kv1.2, and Kv1.6, horizontal dashed line represents the significance threshold (p-value < 0.1, two-sided moderated t test), vertical dashed lines indicate fold change value of 0.4 between Setd5+/+ and Setd5+/. (C) Tissue-specific western blots of Kv1.1 protein content in the SC and the CTX for Setd5+/+ (n = 3) and Setd5+/− mice (n = 3) and (D) their quantification (SC: Setd5+/+, 0.210; Setd5+/−, 0.275, P = 0.226. CTX: Setd5+/+, 0.167; Setd5+/−, 0.235, P = 0.392). P-values are two-tailed Wilcoxon’s signed-rank test. The data underlying this figure can be found in S17 Data and S7 Data. (TIF) [file pbio.3002668.s008.tif]

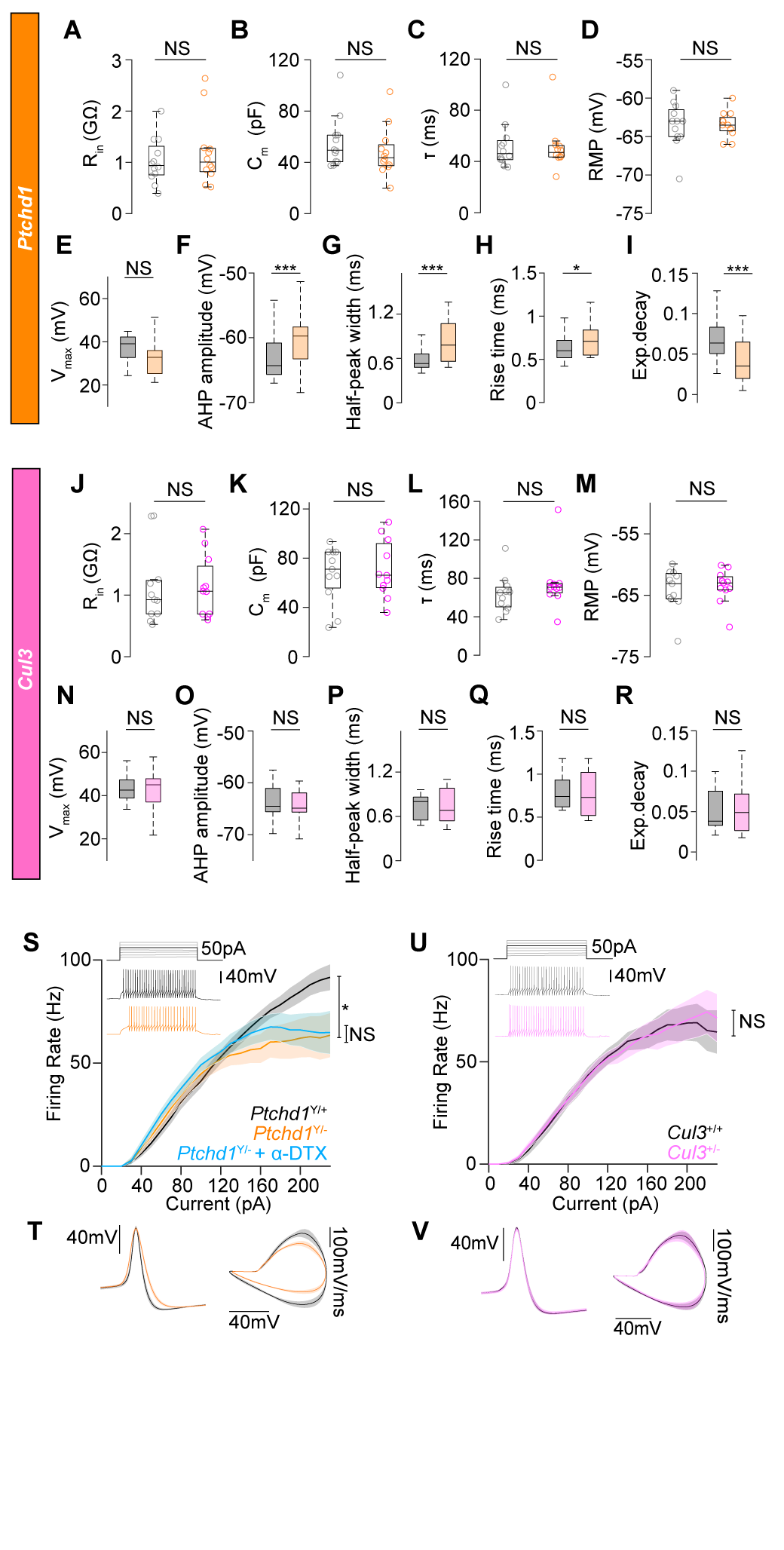

Supplement: S9 Fig — Intrinsic properties of dPAG cells in Cul3 and Ptchd1 animals. (A, J) Input resistance (A, Ptchd1Y/+, 1.03 GΩ; Ptchd1Y/−, 1.20 GΩ, p = 0.623; J, Cul3+/+, 1.12 GΩ; Cul3+/−, 1.14 GΩ, p = 0.896). (B, K) Membrane capacitance (B, Ptchd1Y/+, 55.6 pF; Ptchd1Y/−, 48.2 pF, p = 0.299; K, Cul3+/+, 66.6 pF; Cul3+/−, 70.8 pF, p = 0.896). (C, L) Membrane constant tau (C, Ptchd1Y/+, 52.0 ms; Ptchd1Y/−, 51.2 ms, p = 0.795; L, Cul3+/+, 63.9 ms; Cul3+/−, 74.3 ms, p = 0.212). (D, M), Resting membrane potential (D, Ptchd1Y/+, −63.5 mV; Ptchd1Y/−, −63.5 mV, p = 0.749; M, Cul3+/+, −64.0 mV; Cul3+/−, −63.5 mV, p = 0.844). Action potential kinetics for all spikes generated in the rheobase sweep. (E, N) Action potential amplitude (Vmax) (E, Ptchd1Y/+, 33.5 mV; Ptchd1Y/−, 33.6 mV, p = 0.092; N, Cul3+/+, 43.0 mV, Cul3+/−, 41.9 mV, p = 0.984). (F, O), After-hyperpolarisation (AHP) amplitude (F, Ptchd1Y/+, −63.3 mV; Ptchd1Y/−, −60.7 mV, p < 0.001; O, Cul3+/+, −63.8 mV, Cul3+/−, −64.4 mV, p = 0.296). (G, P) Width at half-peak (G, Ptchd1Y/+, 0.580 ms; Ptchd1Y/−, 0.817 ms, p < 0.001; P, Cul3+/+, 0.717 ms, Cul3+/−, 0.736 ms, p = 0.565). (H, Q) Rise time (H, Ptchd1Y/+, 0.635 ms; Ptchd1Y/−, 0.734 ms, p = 0.011; Q, Cul3+/+, 0.790 ms, Cul3+/−, 0.784 ms, p = 0.399). (I, R) Exponential decay constant (I, Ptchd1Y/+, 0.066; Ptchd1Y/−, 0.043, p < 0.001; R, Cul3+/+, 0.052, Cul3+/−, 0.067, p = 0.867). Points represent Ptchd1Y/+ or Cul3+/+ (grey) and Ptchd1Y/− (orange) or Cul3+/− (pink) dPAG cells. Ptchd1Y/+, n = 4, (A-D) 16 cells, (E-I) 58 spikes; Ptchd1Y/−, n = 4, (A-D) 16 cells, (E-I) 72 spikes. Cul3+/+, n = 3, (J-M) 12 cells, (N-R) 68 spikes; Cul3+/−, n = 3, (J-M) 12 cells, (N-R) 73 spikes. (S) Summary of the relationship between current injection and action potential firing showing a strong reduction in firing in Ptchd1y/− dPAG cells. Effect of genotype without α-DTX: P = 0.046; effect of α-DTX on Ptchd1Y/− firing: P = 0.680. (Ptchd1Y/+, n = 4, 16 cells; Ptchd1Y/−, n = 4, 20 cells; Ptchd1Y/− with α-DTX, n [file pbio.3002668.s009.tif]

## *Ptchd1* gDNA

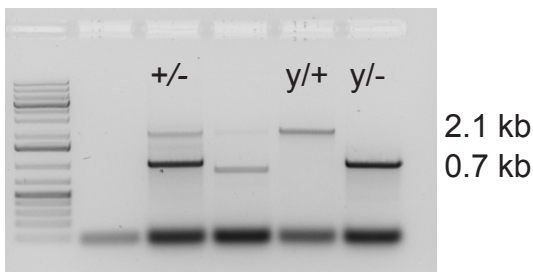

## *Ptchd1* cDNA

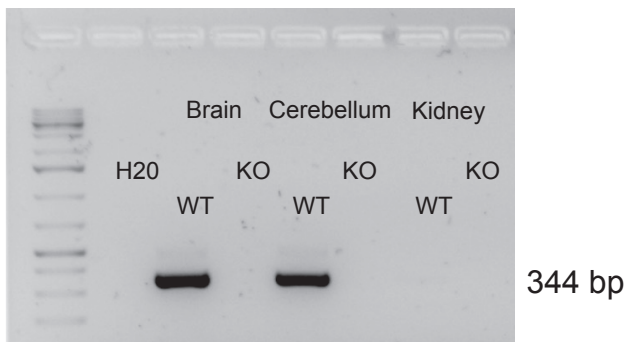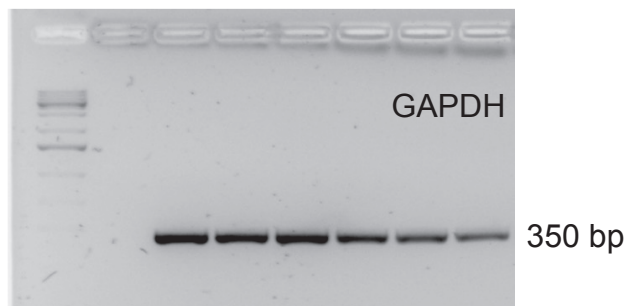

Supplement: S12 Data — (PDF) [file pbio.3002668.s024.pdf]
