# Supplementary material for: Shared behavioural impairments in visual perception and place avoidance across different autism models are driven by periaqueductal grey hypoexcitability in Setd5 haploinsufficient mice
Source: PLoS Biol. 2024 Jun 10;22(6):e3002668. doi: 10.1371/journal.pbio.3002668 (PMC11216578; doi:10.1371/journal.pbio.3002668)
Supplement: S7 Data — (PDF) [file pbio.3002668.s019.pdf]

Figure 6B was cropped from samples 1 and 2.

Figure S8C was cropped from samples 1 and 2.

Figure 6C and S8D quantification was based on samples 1, 2, 3, 4, 5 and 6.

Samples 1 & 2

C1: cortex *Setd5*$^{+/+}$
S1: superior coliculus *Setd5*$^{+/+}$
P1: periaqueductal grey *Setd5*$^{+/+}$
C2: cortex *Setd5*$^{+/-}$
S2: superior coliculus *Setd5*$^{+/-}$
P2: periaqueductal grey *Setd5*$^{+/-}$

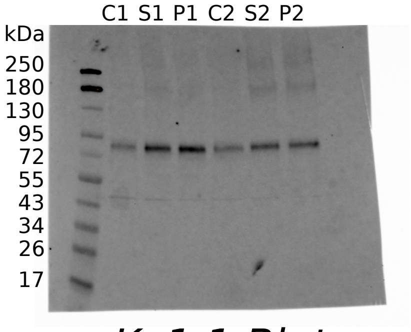

Kv1.1 Blot

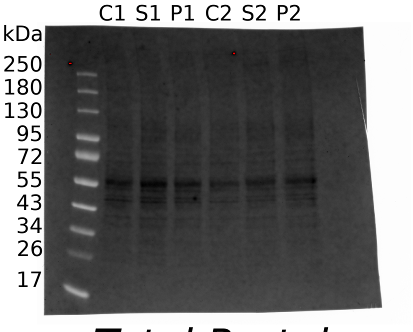

Total Protein

Samples 3 & 4

C3: cortex *Setd5*$^{+/+}$
S3: superior coliculus *Setd5*$^{+/+}$
P3: periaqueductal grey *Setd5*$^{+/+}$
C4: cortex *Setd5*$^{+/-}$
S4: superior coliculus *Setd5*$^{+/-}$
P4: periaqueductal grey *Setd5*$^{+/-}$

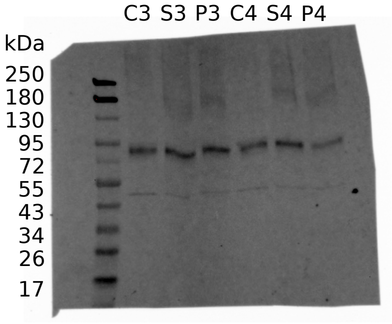

Kv1.1 blot

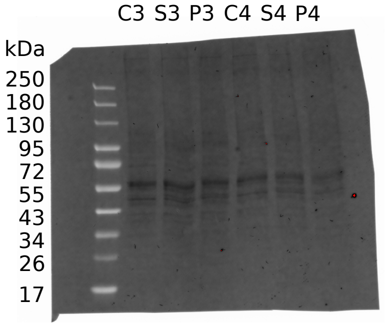

Total Protein

Samples 5 & 6

C5: cortex *Setd5*$^{+/+}$
S5: superior coliculus *Setd5*$^{+/+}$
P5: periaqueductal grey *Setd5*$^{+/+}$
C6: cortex *Setd5*$^{+/-}$
S6: superior coliculus *Setd5*$^{+/-}$
P6: periaqueductal grey *Setd5*$^{+/-}$

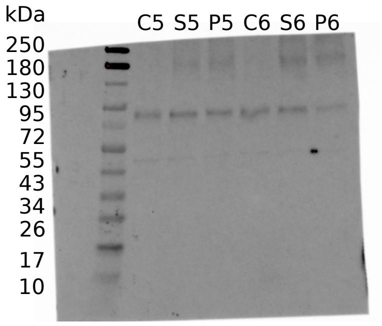

Kv1.1 blot

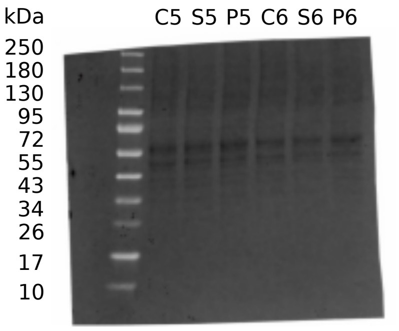

Total Protein
