# Supplementary material for: Shared behavioural impairments in visual perception and place avoidance across different autism models are driven by periaqueductal grey hypoexcitability in Setd5 haploinsufficient mice
Source: PLoS Biol. 2024 Jun 10;22(6):e3002668. doi: 10.1371/journal.pbio.3002668 (PMC11216578; doi:10.1371/journal.pbio.3002668)
Supplement: Attachment — Submitted filename: Reply to Reviewers - Plos Biology 2024.pdf [file pbio.3002668.s031.pdf]

Point-by-point response to the reviewers' comments

We thank the four reviewers for their insightful comments and suggestions. In our revised version of the manuscript, we have added substantial new data and analyses that address the remaining concerns regarding circuit connectivity and molecular aspects of the study.

We believe that our current version addresses the reviewers' concerns by improving clarity and strengthening our previous results. Next, we will evaluate and discuss each reviewer's comments point by point.

Please note that all changes to the text have been marked in red, except for the numbering of figures and the capitalisation of the figure letters. This would have added unnecessary work for the reviewers.

REVIEWS:

Reviewer #1: In this study by Laura E. Burnett et al., the authors explore a recurrent impairment in visual threat perception that is similarly affected in three independent models of Autism Spectrum Disorder (ASD) with different molecular etiologies. They observe a Kv-channel induced dPAG hyperexcitability phenotype, suggesting that the integration and action initiation necessary for adequate perceptual decision-making are disrupted in Setd5 mice. While the topic is intriguing and potentially significant, there are several concerns that need addressing.

Major:

1. The authors assume that the dPAG is a key downstream brain region for the dmSC to generate robust escape responses. Therefore, they should label the postsynaptic neurons in the dPAG that receive innervation from the SC using viral tracing tools and then conduct whole-cell patch-clamp recordings. Moreover, implementing pathway-specific modulation using the appropriate viruses would be advantageous.

Thanks for the suggestion. We have done a similar experiment already, by expressing ChR2 in vGluT2+ dmSC neurons, then patching dPAG neurons while optogenetically activating the dmSC processes (Fig. 5A,B). The fact that we see strong short-latency currents when activating dmSC processes shows that direct, monosynaptic connectivity exists. As shown in a previous report (Evans et al. 2018), we see a similar connectivity strength between dmSC and dPAG. We have now re-analyzed this data to compare the excitability of only dPAG neurons with proven input from the dmSC. Using only these neurons, we observe a similar hypoexcitability phenotype between genotypes. We have added this analysis to Fig. S7K. See below for convenience.

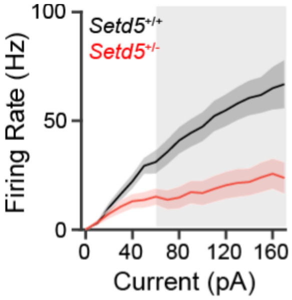

Intrinsic excitability of dPAG cells that receive direct optogenetic input from dmSC neurons (see Fig. 5A,B). Setd5+/+, n = 3, 9 cells; Setd5+/-, n = 4, 12 cells.

We have added the following sentence to the text:

Line 270-273: *"The hypoexcitability phenotype is also pronounced when the same analysis is performed on dPAG cells, which have been shown to receive input from dmSCs (Fig. S7K) using optogenetic activation of dmSC fibres (Fig. 5A, B)."*

Viral tracing experiments, especially retrograde pseudorabies tracing experiments initiated in vGlut2+ neurons in the dPAG, would provide additional evidence for a direct connection. Fortunately, these experiments have already been performed in one of the papers we cite (Evans et al. 2018). In their Supplementary Figure 7, the authors infected VGlut2+ dPAG cells with AAV-TVA and subsequently infected the dPAG with rabies virus expressing mCherry. These experiments show substantial input from the dmSC. For convenience, we have copied that figure to our replies.

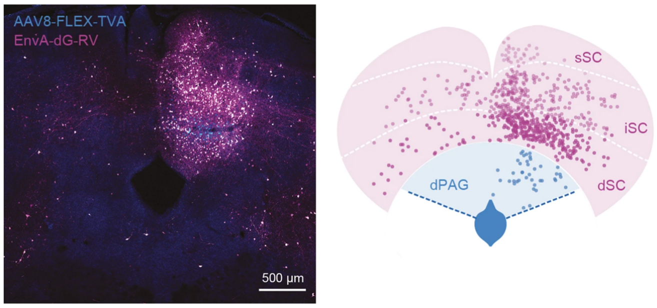

From Evans et al. 2018, Figure S7, panel A. Blue, starter cell position (AAV-TVA), pink, input cells (Pseudorabies)

We agree with the reviewer that pathway-specific modulation would be a very good complementary experiment. In fact, we have tried this in the past by expressing DREADDs in subsets of cells known to be required for escape responses. Unfortunately, we got an unexpected result. Expression of DREADDs alone, without the use of CNO, already had an effect on our control groups of animals (see below).

In brief, we infected either Pvalb-Cre or GRP-Cre animals, known to label cells involved in the escape response, with either AAV-DIO-mCherry or AAV-DIO-hM4Di-mCherry viruses. However, the expression of hM4Di-mCherry alone in either line was sufficient to severely affect their impending avoidance response, with slower responses and a reduction in maximum speed. This was not due to the infection procedure or injection of the saline. AAV-DIO-mCherry control animals had no effect. The deficits were also not due to some anomalies with the DREADD viruses since we tested them and used them successfully in a previous study in the hippocampus (Fredes et al. 2021). Therefore, in the brain structures of interest, the sole expression of DREADDs had side effects, and thus, we preferred not to use this approach for further experiments.

Attempt to selectively disrupt activity in targeted cell populations within the superior colliculus using DREADDs. *Mice were injected with 200nL a cre-dependent virus encoding either the control mCherry fluorophore, or an mCherry-labeled inhibitory DREADD (hM4Di), given 2-3 weeks to recover then injected with saline and exposed to the LER paradigm, then 24 hours later injected with CNO (1 mg kg^{-1}). (Top) Raster plots of Pvalb-cre animals' speed in response to looming stimuli (vertical white lines). Pvalb-Cre;AAV8-DIO-mCherry, n = 2, 6 trials triggered with saline, 5 trials triggered with CNO; Pvalb-Cre;AAV8-DIO-hM4Di, n = 2, 14 trials triggered with saline, 14 trials triggered with CNO. Mean reaction time across trials: mCherry with saline, 0.26s; mCherry with CNO, 0.58s; hM4Di with saline, 2.36s; hM4Di with CNO, 3.59s. Mean maximum escape speed: mCherry with saline, 94.2 cm s^{-1}; mCherry with CNO, 64.3 cm s^{-1}; hM4Di with saline, 55.3 cm s^{-1}; hM4Di with CNO, 52.1 cm s^{-1}. (Bottom) Raster plots of GRP-cre animals' speed in response to looming stimuli (vertical white lines). GRP-Cre;AAV8-DIO-mCherry, n = 4, 4 trials triggered with saline, 4 trials triggered with CNO; GRP-Cre;AAV8-DIO-hM4Di, n = 5, 25 trials triggered with saline, 15 trials triggered with CNO. Mean reaction time across trials: mCherry with saline, 0.32s; mCherry with CNO, 0.58s; hM4Di with saline, 2.95s; hM4Di with CNO, 3.03s. Mean*

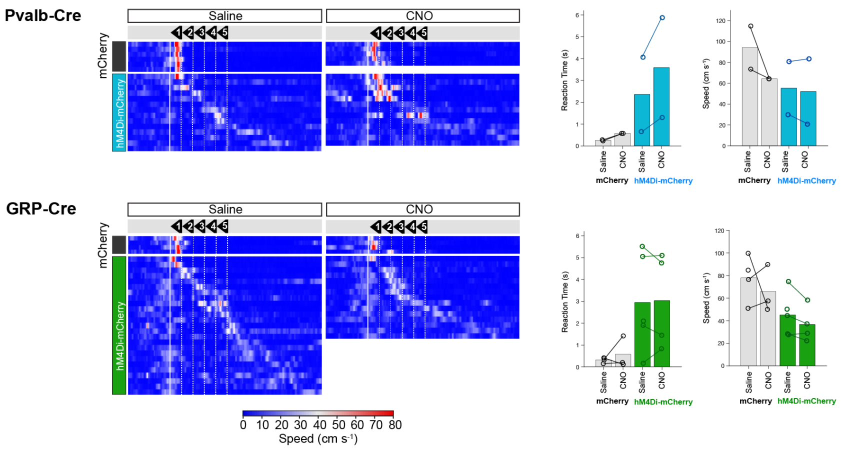

maximum escape speed: mCherry with saline, 78.0 cm s^{-1}; mCherry with CNO, 65.8 cm s^{-1}; hM4Di with saline, 45.1 cm s^{-1}; hM4Di with CNO, 36.7 cm s^{-1}.

2. Figure 4: the authors employ optogenetic stimulation to activate SC neurons, successfully reproducing the delayed LER phenotype in Setd5+/- animals. To comprehensively elucidate the role of the SC, bilateral optogenetic activation is crucial. Additionally, providing a detailed, high-definition confocal micrograph of the injection sites, along with a clear depiction of the virus expression pattern, would be beneficial.

We thank the reviewer for this suggestion, but believe that some clarification of the content of the looming escape response may be required. Mice have been found to have a small bilateral overlap, predominantly in the frontal direction (Holmgren et al. 2021). Given the retinotopic organisation, the majority of each superior colliculus (SC) receives information from a single eye, particularly in the area where looming stimuli are presented. This indicates that the response to a looming threat in mice is primarily due to the activation of one SC. Furthermore, it has been shown that both SCs inhibit each other via GABAergic projections (Essig, Hunt, and Felsen 2021). Thus, simultaneous activation of both SCs would introduce further artificiality into the experimental paradigm, making the results less interpretable in the context of understanding the looming escape response. Thus, we don't think that in the context of our behavioural paradigm, these experiments would shed light on the SC's role.

As requested by the reviewer, we have added a panel to supplementary figure 6 showing the ChR2 expressing vGlut2+ dmSC projections to the dPAG. We have added these panels also below for convenience.

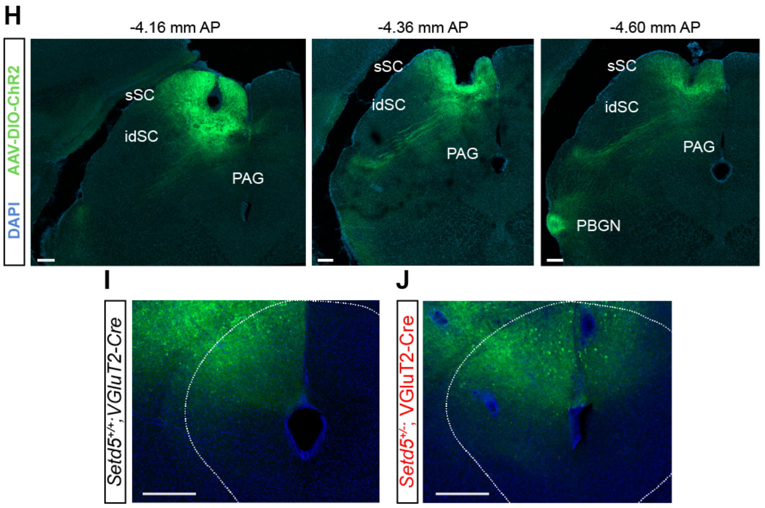

*Additional confocal micrographs of AAV-DIO-ChR2 viral expression pattern at different anterior-posterior (AP) positions and optic fibre placement above the idSC (H), coordinates are in mm and from bregma. In addition, **c**lose-up to the dPAG in Setd5$^{+/+}$ (I) and Setd5$^{+/-}$ (J) (scale bar: 200 µm).*

3. Figure 5: the referenced transcriptomic data suggests upregulation of Setd5+/- Kv channels, and the application of -DTX reversed the hypoexcitability phenotype in Setd5+/- cells. However, the authors found no changes in Kv channels within the PAG. The authors attribute this discrepancy to homeostatic misregulation but fail to provide supporting references.

Perhaps the review might have missed the sentence in the discussion section. We provide references to three possible mechanisms of how homeostatic missregulation might be affecting Kv channel conductances:

Line XX: "Given that the expression levels of Kv channels remain unaltered (Fig. 5K, Fig. S7), the causes of the physiological hypoexcitability phenotype probably arise through homeostatic misregulation, either by localisation (Zbili et al. 2021)*, post-translational modifications* (Winklhofer et al. 2003)*, or interactions with auxiliary subunits* (Niespodziany et al. 2019)*."*

4. Figure 6: the authors attempt to mitigate delayed LER and place avoidance by implanting a drug delivery cannula in close proximity to the dPAG. However, considering the superior location of the SC to the PAG, how can one ensure the prevention of any potential damage to the SC?

As the reviewer correctly pointed out, there is some damage across the layers of the SC. However, it is worth noting that the damage is relatively minor. The cannulas are similar in size to the small optical fibres used in optogenetics (0.21 mm outer diameter), which minimises injury. Notably, the animals still respond to the loom in the same way as those without the procedure. In WT siblings, they responded immediately after the loom and in Setd5(+/-), they only stopped responding before the escape in control conditions. This suggests that their visual pathway remains largely intact. The most parsimonious explanation is that the injury is at a different retinotopic location. The looming stimulus is presented from the ceiling, which would be perceived by the mouse from more medial coordinates in the SC that remain undamaged.

5. The uppercase and lowercase letters in the result figures within the manuscript do not correspond to those in the figure captions, causing considerable confusion.

Corrected

Reviewer #2: The manuscript by Burnett et al. has provided well-controlled behavioral data on the conserved impairment of visual perception and place avoidance in 3 autism models (Setd5+/-, Cul3+/- and Ptchd1Y/-). In addition, it has revealed that the behavioral deficits are causally linked to a potassium channel (Kv1)-mediated hypoexcitability in dorsal periaqueductal grey (dPAG) of Setd5+/- mice. While the experiments are well designed and executed, there are a few concerns that need to be addressed.

1. The identified molecular mechanism (Kv1-mediated hypoexcitability of dPAG neurons) does not apply to Cul3+/- and Ptchd1Y/- mice, suggesting that it is not a common basis of perceptual impairment across autism models. The title is confusing and misleading, and should be changed. The abstract should clarify that the mechanism is limited to only one autism model.

We apologise for the confusion and have amended the text accordingly. It is important to note that we believe the recurrent nature of the behavioural impairment is key to the study. As with people with ASD, this shows a robust, quantifiable and reproducible behavioural criterion. Given the underlying molecular differences between the animals, we expected that the underlying physiological conditions would differ. Although outside the core symptoms typically used for diagnosis, the fact that the visuomotor pathways are well-defined allowed us to perform a deep dissection of the underlying dysfunction in at least one of these models.

New title: *"Common behavioural impairments in visual perception and place avoidance across autism models are causally linked in the haploinsufficiency model of intellectual disability Setd5"*

Added sentence to the abstract: *"Furthermore, we show that different molecular mechanisms converge on similar behavioural phenotypes by demonstrating that the autism models Cul3 and Ptchd1, despite having similar behavioural phenotypes, differ in their functional and molecular alteration."*

2. More discussions on the different mechanisms for the conserved LER deficits across autism models are needed.

Thank you for raising this point. There are indeed several circuit dysfunctions that could produce similar behavioural phenotypes. To streamline our results, we thought it would be beneficial to be more focused, but in retrospect, we agree with the reviewers' point that a more careful comparison would be beneficial. First, the most parsimonious interpretation would be that visual transmission is impaired. Since low-contrast loom stimuli elicit weaker responses, weaker visual transmission would have a similar result. This is why we tested visual responses in Setd5 animals. We have added similar experiments for Cul3 and Ptchd1 (New Supplementary Fig. 5, see below for conveniene), which we now use as an argument to define other theoretically possible mechanisms, which can include, e.g., weaker synaptic transmission or imbalance in the excitation/inhibition of downstream networks.

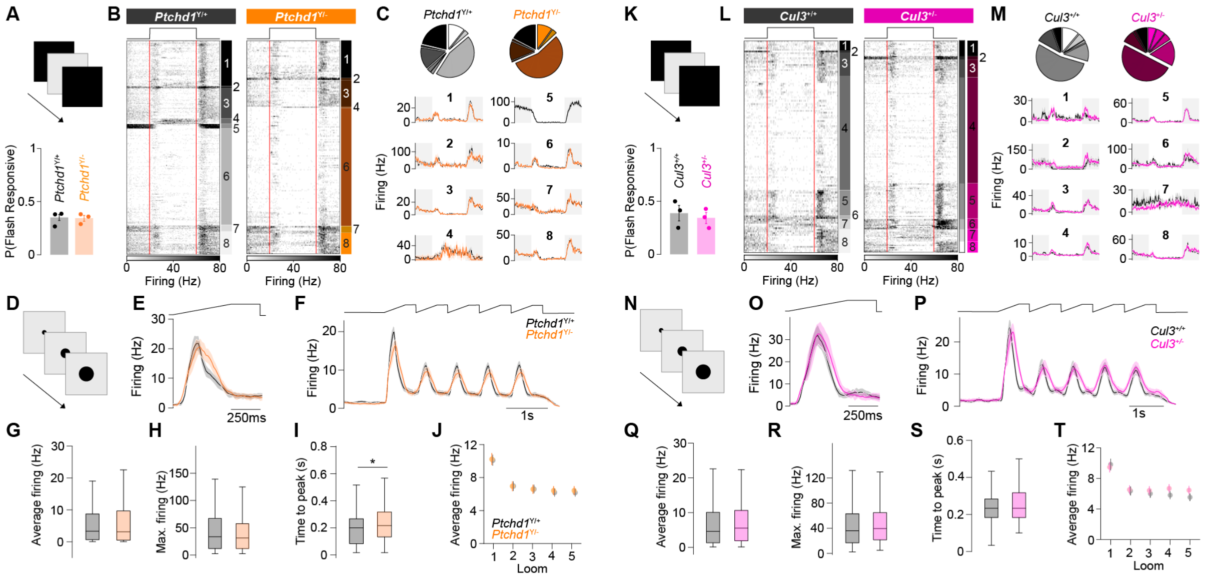

Fig. S5. Visual response properties for Cul3 and Ptchd1. ***A***, *Schematic of visual stimulus & proportion of sSC cells that are flash responsive (Ptchd1*$^{Y/+}$*: 0.350; Ptchd1*$^{Y/-}$*: 0.343, P = 0.857, two-way t-test).* ***B***, *Sorted raster plot of neural responses to a single flash stimulus. Vertical red dotted lines indicate the onset and offset of the flash stimulus.* ***C***, *Proportion of cells in each cluster (top). Mean ± s.e.m. of the flash responses in each cluster (traces).* ***D***, *Schematic of the loom stimulus.* ***E***, *Mean ± s.e.m. response of all of the first loom stimuli and (****F****) of the 5 consecutive loom stimuli for Ptchd1*$^{Y/+}$ *(black) and Ptchd1*$^{Y/-}$ *(orange) sSC units.* ***G-I***, *Summary of mean, maximum and time-to-peak firing (****G****, P = 0.461;* ***H***, *P = 0.523;* ***I***, *P = 0.049).* ***J***, *Average firing to a single loom across the 5-loom stimulus (p = 0.758).* ***K-T***, *as for* ***A-J*** *but for the Cul3 mouse model.* ***K***, *Proportion of sSC cells that are flash responsive (Cul3*$^{+/+}$*: 0.388; Cul3*$^{+/-}$*: 0.342, P = 0.638, two-way t-test).* ***L***, *Sorted raster plot of neural responses to a single flash stimulus. Vertical red dotted lines indicate the onset and offset of the flash stimulus.* ***M***, *Proportion of cells in each cluster (top). Mean ± s.e.m. of the flash responses in each cluster (traces).* ***N***, *Schematic of the loom stimulus.* ***O***, *Mean ± s.e.m. response of all of the first loom stimuli and (****P****) of the 5 consecutive loom stimuli for Cul3*$^{+/+}$ *(black) and Cul3*$^{+/-}$ *(magenta) sSC units.* ***Q-S***, *Summary of mean, maximum and time-to-peak firing (****Q****, P = 0.222;* ***R***, *P = 0.214;* ***S***, *P = 0.879).* ***T***, *Average firing to a single loom across the 5-loom stimulus (p = 0.267). Ptchd1*$^{Y/+}$*, n = 3, 263 sSC cells; Ptchd1*$^{Y/-}$*, n = 3, 202 sSC cells. Cul3*$^{+/+}$*, n = 3, 205 sSC cells; Cul3*$^{+/-}$*, n = 3, 204 sSC cells. P-values: Wilcoxon's test, p-values: two-way repeated measures ANOVA.*

We have added the following sentence and paragraph to the text:

Line 216-218: "*Similarly, no differences were observed when comparing the visual response properties of Cul3 and Ptchd1 models (Fig. S5).*"

Lines 439-458: "This is not a trivial task, as several possible changes could lead to delays in LER. The simplest explanation for these behavioural impairments is abnormalities in early visual processing. For example, if salient, high-contrast loom stimuli are relayed as low-contrast looms to dPAG neurons, these would be weakly activated, leading to delayed LERs (Evans et al. 2018). Other mechanisms that could lead to weaker dPAG activation include changes in synaptic transmission, e.g. from dmSC to dPAG, imbalance in the excitatory-inhibitory ratio of the circuits involved (Gao and Penzes 2015), abnormal development of brain connectivity (Belmonte et al. 2004), or reduced excitability in dPAG neurons, among many others. Therefore, the exact molecular changes are likely to be model-dependent. We show that in all models tested, early visual processing (Figs. S4, S5) and most intrinsic properties (Figs. S7, S9) remain unaffected. The main difference observed is the dPAG hypoexcitability phenotype, which we were able to causally link to LER in the Setd5 model (Fig. 5). In addition, we were able to correlate a similar hypoexcitability phenotype in the dPAG with LER in Ptchd1 animals, albeit through a different molecular mechanism. This already shows that different molecular perturbations lead to similar behavioural phenotypes, as further demonstrated by the weaker LER deficits in Cul3 animals that are independent of the intrinsic properties of the dPAG (Fig. S9). This level of dissection can lead to important insights that may reveal therapeutic targets, as envisioned by precision medicine approaches (Kostic and Buxbaum 2021). Specifically, our study shows that in the Setd5 haploinsufficient model, these behaviours are not necessarily developmental, as they can be pharmacologically ameliorated in adulthood."

3. Fig. 5K does not clearly indicate the significant alteration of KCNA1 and KCNA2 in Setd5+/- mice. Are they "differential protein levels" (Line 117) or differential mRNA levels? Are they from the same kind of Setd5+/- mice as what is used here? What cell types were used in Fig. 5K? The authors need to confirm the changes in the expression of Kv1.1, Kv1.2, Kv1.6 and other K channels (targets of a-DTX) in pPAG cells of Setd5+/- mice.

We apologise for the misunderstanding. Figure 5K, which is now part of a new Fig. 6, is from proteomics data from ~ 1 mm^3 tissue samples of PAG and is not cell-type specific. We believe this is a good approximation as we observe the hypoexcitability phenotype across the measured cell types. These samples are from identical animals used in behavioural experiments. Furthermore, the work we cite on *Setd5* mRNA levels was done in our institute, from the same animals, and in the same facility (Deliu et al. 2018). The work we cite was performed in our institute by our co-author Gaia Novarino. The difference in mRNA was only visible during development (E9), published in (Deliu et al. 2018). In contrast to the embryonal data, we don't see any change in adulthood, so our interpretation is that the channels have to be modulated differently, as argued previously:

Line 426-429: "Given that the expression levels of Kv channels remain unaltered (Fig. 5K, Fig. S7), the causes of the physiological hypoexcitability phenotype probably arise through homeostatic misregulation, either by localisation(Zbili et al. 2021), post-translational modifications(Winklhofer et al. 2003), or interactions with auxiliary subunits(Niespodziany et al. 2019)."

In the volcano plot, the horizontal dashed line represents the significance threshold (P-value < 0.1) from a two-sided moderated *t*-test, while the vertical dashed lines indicate fold change values greater or less than 0.4 between *Setd5*$^{+/+}$ and *Setd5*$^{+/-}$. Thus, changes in Kv channel protein levels are small and show a slight decrease in *Setd5*$^{+/-}$ compared to their WT siblings (*Setd5*$^{+/+}$). This is also seen in our western blot analysis (New Fig. 6B-C).

As the only known Kv channels targeted by a-DTX are Kv1.1, Kv1., and Kv1.6, we can therefore confirm that there are no changes in the protein levels of any a-DTX-sensitive Kv channels that could account for an increase in hypoexcitability. a-DTX also inhibits ASIC channels, pH-sensing channels that are Na-permeable. Blocking these ASIC channels would have the exact opposite effect. Therefore, we excluded these from our analysis.

Note that we have now separated the volcano plot from Fig. 5 and combined it with the other experiments quantifying Kv expression levels in a new Fig. 6. We believe this will help to separate the two arguments presented more clearly.

4. The conclusion will be more convincing if the authors perform patch clamp recordings to compare Kv1.1 channel currents in pPAG cells of Setd5+/+ vs. Setd5+/- mice.

Thank you for your suggestion. We have performed the requested experiments. We patched dPAG neurons and voltage clamping them with Nav blockers and compared the difference between the I-V curves with and without a-DTX. As expected from the current-firing relationship, we observed a marked increase in Kv current in Setd5(+/-) compared to their WT siblings (see below). We have added this plot to Fig. 5 and below for convenience.

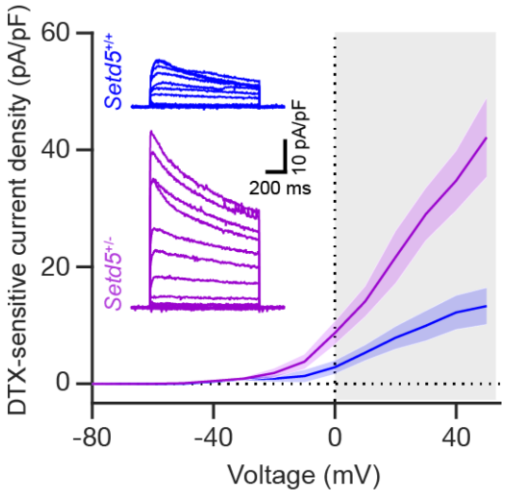

Quantification of a-DTX-sensitive currents in dPAG neurons. *dPAG neurons in Setd5(+/+) and Setd5(+/-) slices were patched with QX-314 in the pipette solution to block all inward Na currents. Next, current steps of different magnitudes were applied first without and then with a-DTX in the extracellular solutions. The differences were then analysed as a proxy for a-DTX-sensitive currents. Inset: Example of a-DTX-sensitive currents in Setd5(+/+) and Setd5(+/-) dPAG neurons.*

We have added the following sentence to the text:

Line 298-300: "*Accordingly, we measured a drastically increase* ***α****-DTX sensitive outward current in Setd5+/- dPAG cells (Fig. 5I).*"

5. Fig. 5G shows no effect of a-DTX on firing rate of Setd5+/+ mice, why is that? Blocking K+ channels is expected to increase firing rate in WT mice too.

We do see a slight increase in firing rate at higher current injections (see below, black WT, blue WT+DTX, from panel 5G).

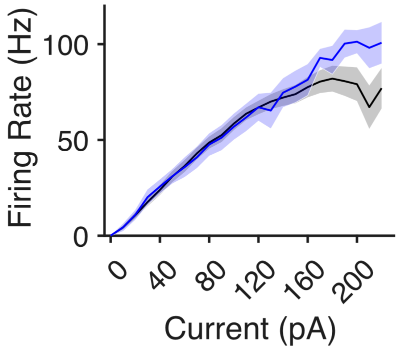

Current injection in WT dPAG cells. Data from Fig. 5G. Black: Setd5$^{+/+}$, 18 cells; Blue: Setd5$^{+/+}$ with a-DTX, 12 cells.

However, we were also surprised by the comparatively small contribution since it means that the Kv channels involved should be largely silent. The simplest explanation for the changes in Kv expression doesn't seem to hold, because we don't see any major changes in protein levels. Thus, the remaining explanations would be that most of the Kv channels are either not located in the initial segment of the axon, as has recently been shown to be a mechanism for homeostatic regulation (Zbili et al. 2021), or they have a lower conductance, e.g., if modulated (Boland and Jackson 1999), as has been shown previously. This leads to the interesting hypothesis that animals may actively use the modulation of Kv conductances in the dPAG to control behavioural adaptations. This is now discussed directly:

Line 377-382: Interestingly, it is not the level of expression of the channel that appears to be important, as no difference was found between Setd5$^{+/-}$ and Setd5$^{+/+}$ at the protein level (Fig. 5K). This indicates that Kv1 channels may be generally inactive in wild-type animals, as evidenced by the lack of increase in excitability in Setd5$^{+/+}$ animals during a-DTX application, and suggests that changing the conductance of Kv channels may be a mechanism for behavioural adaptation to threatening stimuli, such as imminent suppression of escape(Lenzi et al. 2022).

Line 426-429: Given that the expression levels of Kv channels remain unaltered (Fig. 5K, Fig. S8), the causes of the physiological hypoexcitability phenotype probably arise through homeostatic misregulation, either by localisation (Zbili et al. 2021), post-translational modifications (Winklhofer et al. 2003), or interactions with auxiliary subunits (Niespodziany et al. 2019).

6. Line 213, Fig. 4j should be changed to Fig. S4j. Line 215, Fig. 4o,p should be changed to Fig. S4o,p. Line 251, Fig. 4 should be changed to Fig. 5.

Corrected

References

Belmonte, Matthew K., Greg Allen, Andrea Beckel-Mitchener, Lisa M. Boulanger, Ruth A. Carper, and Sara J. Webb. 2004. “Autism and Abnormal Development of Brain Connectivity.” *The Journal of Neuroscience: The Official Journal of the Society for Neuroscience* 24 (42): 9228–31.

Boland, L. M., and K. A. Jackson. 1999. “Protein Kinase C Inhibits Kv1.1 Potassium Channel Function.” *The American Journal of Physiology* 277 (1): C100–110.

Deliu, Elena, Niccolo Arecco, Jasmin Morandell, Christoph P. Dotter, Ximena Contreras, Charles Girardot, Eva-Lotta Käsper, et al. 2018. “Haploinsufficiency of the Intellectual Disability Gene SETD5 Disturbs Developmental Gene Expression and Cognition.” *Nature Neuroscience* 21 (12): 1717–27.

Essig, Jaclyn, Joshua B. Hunt, and Gidon Felsen. 2021. “Inhibitory Neurons in the Superior Colliculus Mediate Selection of Spatially-Directed Movements.” *Communications Biology* 4 (1): 719.

Evans, Dominic A., A. Vanessa Stempel, Ruben Vale, Sabine Ruehle, Yaara Lefler, and Tiago Branco. 2018. “A Synaptic Threshold Mechanism for Computing Escape Decisions.” *Nature* 558 (7711): 590–94.

Fredes, Felipe, Maria Alejandra Silva, Peter Koppensteiner, Kenta Kobayashi, Maximilian Joesch, and Ryuichi Shigemoto. 2021. “Ventro-Dorsal Hippocampal Pathway Gates Novelty-Induced Contextual Memory Formation.” *Current Biology: CB* 31 (1): 25–38.e5.

Gao, R., and P. Penzes. 2015. “Common Mechanisms of Excitatory and Inhibitory Imbalance in Schizophrenia and Autism Spectrum Disorders.” *Current Molecular Medicine* 15 (2): 146–67.

Holmgren, Carl D., Paul Stahr, Damian J. Wallace, Kay-Michael Voit, Emily J. Matheson, Juergen Sawinski, Giacomo Bassetto, and Jason Nd Kerr. 2021. “Visual Pursuit Behavior in Mice Maintains the Pursued Prey on the Retinal Region with Least Optic Flow.” *eLife* 10 (October). https://doi.org/10.7554/eLife.70838.

Kostic, Ana, and Joseph D. Buxbaum. 2021. “The Promise of Precision Medicine in Autism.” *Neuron* 109 (14): 2212–15.

Lenzi, Stephen C., Lee Cossell, Benjamin Grainger, Sarah F. Olesen, Tiago Branco, and Troy W. Margrie. 2022. “Threat History Controls Flexible Escape Behavior in Mice.” *Current Biology: CB* 32 (13): 2972–79.e3.

Niespodziany, Isabelle, Brice Mullier, Véronique Marie André, Philippe Ghisdal, Eric Jnoff, David Moreno-Delgado, Dominique Swinnen, Zara Sands, Martyn Wood, and Christian Wolff. 2019. “Discovery of a Small Molecule Modulator of the Kv1.1/Kvβ1 Channel Complex That Reduces Neuronal Excitability and in Vitro Epileptiform Activity.” *CNS Neuroscience & Therapeutics* 25 (4): 442–51.

Winklhofer, M., K. Matthias, G. Seifert, M. Stocker, S. Sewing, T. Herget, C. Steinhäuser, and S. Saaler-Reinhardt. 2003. “Analysis of Phosphorylation-Dependent Modulation of Kv1.1 Potassium Channels.” *Neuropharmacology* 44 (6): 829–42.

Zbili, Mickaël, Sylvain Rama, Maria-José Benitez, Laure Fronzaroli-Molinieres, Andrzej Bialowas, Norah Boumedine-Guignon, Juan José Garrido, and Dominique Debanne. 2021. “Homeostatic Regulation of Axonal Kv1.1 Channels Accounts for Both Synaptic and Intrinsic Modifications in the Hippocampal CA3 Circuit.” *Proceedings of the National Academy of Sciences of the United States of America* 118 (47). https://doi.org/10.1073/pnas.2110601118.
