# Supplementary material for: Shared behavioural impairments in visual perception and place avoidance across different autism models are driven by periaqueductal grey hypoexcitability in Setd5 haploinsufficient mice
Source: PLoS Biol. 2024 Jun 10;22(6):e3002668. doi: 10.1371/journal.pbio.3002668 (PMC11216578; doi:10.1371/journal.pbio.3002668)
Supplement: Attachment — Submitted filename: Plos Biology _ Revisions.pdf [file pbio.3002668.s032.pdf]

We are grateful to the reviewer for their detailed comments and for highlighting issues that require further clarification. We have addressed the concerns point by point below:

1. In my last review, I pointed out that the identified molecular mechanism (Kv1-mediated hypoexcitability of dPAG neurons) does not apply to Cul3+/- and Ptchd1Y/- mice, suggesting that it is not a common basis of perceptual impairment across autism models, so the original title (Recurrent impairments in visual perception and place avoidance across autism models are causally linked in the haploinsufficiency model of intellectual disability Setd5) is misleading. Now the new title "Common behavioural impairments in visual perception and place avoidance across autism models are causally linked in the haploinsufficiency model of intellectual disability Setd5 '' has little change and is still problematic. While the behavioral phenotypes are similar among the three autism models, molecular mechanisms are distinct. Thus, it lacks evidence showing that they are "causally linked". The title has to be toned down.

The term "causally linked" in the title may have been misleading. We apologise for this misunderstanding. We did not mean to imply that all three models exhibit the same behavioural impairments due to a common molecular mechanism, but rather that the *Setd5* model provides a direct link between common behavioural impairments and lineage-specific neurophysiological changes in the dorsal PAG. We thought we had addressed the concerns directly in our revised abstract by emphasising that the effects are specific to *Cul3* and *Ptchd1*.

"Furthermore, we show that different molecular mechanisms converge on similar behavioural phenotypes by demonstrating that the autism models Cul3 and Ptchd1, despite having similar behavioural phenotypes, differ in their functional and molecular alterations".

Regarding the title, we think it is important to describe that the behavioural phenotypes are shared between the models. This is important because it is particularly difficult to find such common quantifiable differences across ASD models. Therefore, we suggest changing the title to:

"Shared behavioural impairments in visual perception and place avoidance across different autism models are driven by periaqueductal grey hypoexcitability in Setd5 haploinsufficient mice."

This title also de-emphasises the role of specific Kv channels, which we believe is a secondary finding as discussed below, and emphasises the circuit-level dissection of our work.

2. In my last review, I pointed out that Fig. 5K (now Fig. 6A) does not clearly indicate the significant alteration of KCNA1 and KCNA2 in Setd5+/- mice and the authors need to confirm the changes in the expression of Kv1.1, Kv1.2, Kv1.6 (targets of a-DTX) in pPAG cells of Setd5+/- mice. Now Fig. 6B &C shows only one protein, Kv1.1 (KCNA1), which actually does not show a significant change in the volcano plot (Fig. 6A). With 3 samples in each group of the WB experiments (no labeling of group identities), the statistical conclusion (ns) is unconvincing. In addition, Fig. 6D has no quantification. Why Kv1.6 (KCNA6) was not tested? The direct evidence supporting the alteration of Kv1 channels in Setd5+/- phenotypes is lacking.

We apologize for not having understood the first request to confirm changes in expression with other means. We understood that the proteomic data was not clearly presented.

In our study, we show that there is no statistically significant change in expression using the most sensitive measure in the field - proteomics, for which we have 6 samples at a time. These are more sensitive results than any western-blot analysis, which is always semi-quantitative at most. Specifically, using our proteomics approach, we do not see statistically significant changes in the expression of Kv1.1, Kv1.2, or Kv1.6. For all tests, regulated protein groups were defined as those with a significant P-value and a log2 ratio greater than 5 % of control-to-average-control ratios, a standard in the field (See Fig. 6a). This proteomics data is truly quantitative, highly reproducible across samples and less prone to failure compared to classical biochemistry techniques, like western blots. We would like to draw attention to the following article which makes this very point:

“The incongruity of validating quantitative proteomics using Western blots” (Mehta et al. 2022). The authors state in their introduction: *“nearly every researcher who uses proteomics technologies has at one time or another been asked by a reviewer to provide ‘western blot validation’ of their mass-spectrometry-based protein abundance data. We believe that this request demonstrates a lack of awareness amongst the plant biology community about the extraordinary improvements in cost, sensitivity and reliability that the field of mass-spectrometry-based proteomics has made in recent years.“* Given the advancement of the field and the state-of-the-art mass-spectrometry facility we have at ISTA, demonstrating that there is no change in Kv channel expression using a lesser method (i.e. Western blots) would not further clarify the mechanisms and wouldn't change any of our claims, but would add several months of breeding and antibody validation. The Western blot analysis that we have added to Figure 6 was previously in the first submission as a supplementary figure, we have simply moved it to the main figures because we have restructured the figures for readability. We apologise for not pointing this out in our initial response, given the sensitivity of proteomics we didn't initially understand the request.

Further quantitative validation can be taken from mRNA Seq experiments in adult mice (Deliu et al. 2018) (albeit in a different brain region). These show no changes at the mRNA level of any of the Kv channels, indicating that there is no global change in expression. This is further supported by our proteomic samples in SC and cortex.

Importantly, the mechanism underlying the increase in DTX-sensitive currents, either regulation of expression or modulation of conductances, is not the focus of our work. In our study, we (i) established a behavioural paradigm showing recurrent behavioural impairments in ASD models, (ii) dissected the brain circuits involved, (iii) defined the physiological origin, (iv) pinpointed the molecular culprit (Kv channels), and (v) rescued the behavioural deficits. In a follow-up study, we are dissecting the exact mechanism that regulates the change in Kv conductance, but this is beyond the scope of this study. Therefore, we have also changed the title to emphasise the circuit-level dissection rather than the molecular mechanism.

3. In my last review, I asked why Fig. 5G shows no effect of α-DTX on firing rate in WT mice, since blocking K+ channels is expected to increase firing rate. The authors responded (p16-17) that "most of the Kv channels are either not located in the initial segment of the axon" or "they have a lower conductance, e.g., if modulated (what does this mean?)". These speculations are contradictory to the finding that "Kv1.1 is involved in the homeostatic control of firing [33,34] through modulation of the axon initial segment (AIS)[35]". The result raises concerns on the conclusion that DTX-sensitive Kv1 channels cause hypo-excitability in Setd5+/- mice.

Although we also expected the firing rate to increase, it does so only at the strongest current injections, when maximum firing frequencies are reached and control frequencies begin to decline. See below:

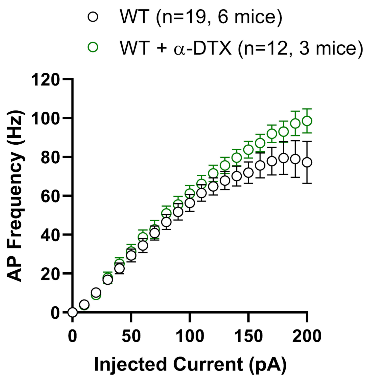

This is consistent with our DTX-sensitive current experiments, where we found large DTX-sensitive current densities only at very positive membrane potentials above 20 mV, whereas *Setd5* mice showed enhanced DTX-sensitive currents already around membrane potentials of 0 mV. Thus, the activity-dependent suppression of neuronal excitability that occurs naturally in WT dPAG neurons may occur at lower stimulation intensities in *Setd5* mice due to enhanced DTX-sensitive currents. Since the membrane properties of WT and *Setd5* dPAG neurons were not different, the larger DTX-sensitive currents in the mutant mice would be expected to have a greater impact on neuronal excitability.

In our previous response as to why the excitability of wild-type animals is not affected by DTX application, we should have emphasised that Kv channels in wild type animals might be scarce at the axon initial segment due to intact homeostatic mechanisms such as those described in Zbili et al. (2021). These regulatory mechanisms might be affected in the *Setd5* animals resulting in the aberrant localisation of these channels to the axon initial segment. Therefore, we do not think these speculations are contradictory to the finding that "*Kv1.1 is involved in the homeostatic control of firing [33,34] through modulation of the axon initial segment (AIS)[35]*". It is also possible that Kv channels are differently distributed within different subcellular compartments or cell populations, which would not be detectable by our bulk sequencing of the PAG. These changes would all result in a change in the effective number of Kv channels in the cell membrane without a noticeable effect on overall expression levels.

Alternatively, without any changes in channel localisation or abundance, it has been shown that the gating properties of Kv channels can be regulated by factors such as activity levels and neuromodulators ((Siegelbaum, Camardo, and Kandel 1982), and that Kv1.1 conductance can be modulated by other factors, such as Protein Kinase C (Boland and Jackson 1999). These were the types of modulatory mechanisms we were referencing in our initial response.

We have refined our discussion of the various ways in which factors independent of overall Kv channel abundance might be affected, and emphasise that these hypotheses require further investigation:

“Given that the expression levels of Kv channels remain unaltered (Fig. 5K, Fig. S8), the causes of the physiological hypoexcitability phenotype probably arise through homeostatic misregulation, either by altered cellular or subcellular channel localisation[33], post-translational modifications[46], or interactions with auxiliary subunits[47]. Through these mechanisms, neuronal excitability could be modified by changing the effective number of Kv channels present in the membrane, or by modulating the gating or conductance of the channels, without changing the overall expression of the channels. Further experiments are required to determine the validity of these hypotheses.”

However, it is beyond the scope of our work to test them thoroughly and draw conclusions about these regulatory mechanisms.

Yours sincerely,

Maximilian Jösch

Boland, L. M., and K. A. Jackson. 1999. “Protein Kinase C Inhibits Kv1.1 Potassium Channel Function.” *The American Journal of Physiology* 277 (1): C100–110.

Deliu, Elena, Niccolo Arecco, Jasmin Morandell, Christoph P. Dotter, Ximena Contreras, Charles Girardot, Eva-Lotta Käsper, et al. 2018. “Haploinsufficiency of the Intellectual Disability Gene SETD5 Disturbs Developmental Gene Expression and Cognition.” *Nature Neuroscience* 21 (12): 1717–27.

Mehta, Devang, Amir H. Ahkami, Justin Walley, Shou-Ling Xu, and R. Glen Uhrig. 2022. “The Incongruity of Validating Quantitative Proteomics Using Western Blots.” *Nature Plants* 8 (12): 1320–21.

Siegelbaum, S. A., J. S. Camardo, and E. R. Kandel. 1982. “Serotonin and Cyclic AMP Close Single K+ Channels in Aplysia Sensory Neurones.” *Nature* 299 (5882): 413–17.
